# Chromosome-level assemblies from diverse clades reveal limited structural and gene content variation in the genome of *Candida glabrata*

**DOI:** 10.1186/s12915-022-01412-1

**Published:** 2022-10-08

**Authors:** Marina Marcet-Houben, María Alvarado, Ewa Ksiezopolska, Ester Saus, Piet W. J. de Groot, Toni Gabaldón

**Affiliations:** 1 Barcelona Supercomputing Centre (BSC-CNS), Plaça Eusebi Güell, 1-3, 08034 Barcelona, Spain; 2 Institute for Research in Biomedicine (IRB Barcelona), The Barcelona Institute of Science and Technology, Baldiri Reixac, 10, 08028 Barcelona, Spain; 3 Regional Center for Biomedical Research, University of Castilla-La Mancha, E-02008 Albacete, Spain; 4 Castilla-La Mancha Science & Technology Park, E-02006 Albacete, Spain; 5 Catalan Institution for Research and Advanced Studies (ICREA), Barcelona, Spain; 6 Centro Investigación Biomédica En Red de Enfermedades Infecciosas, Barcelona, Spain

**Keywords:** *Candida glabrata*, Genomics, Pan-genome, Adhesins

## Abstract

**Background:**

*Candida glabrata* is an opportunistic yeast pathogen thought to have a large genetic and phenotypic diversity and a highly plastic genome. However, the lack of chromosome-level genome assemblies representing this diversity limits our ability to accurately establish how chromosomal structure and gene content vary across strains.

**Results:**

Here, we expanded publicly available assemblies by using long-read sequencing technologies in twelve diverse strains, obtaining a final set of twenty-one chromosome-level genomes spanning the known *C. glabrata* diversity. Using comparative approaches, we inferred variation in chromosome structure and determined the pan-genome, including an analysis of the adhesin gene repertoire. Our analysis uncovered four new adhesin orthogroups and inferred a rich ancestral adhesion repertoire, which was subsequently shaped through a still ongoing process of gene loss, gene duplication, and gene conversion.

**Conclusions:**

*C. glabrata* has a largely stable pan-genome except for a highly variable subset of genes encoding cell wall-associated functions. Adhesin repertoire was established for each strain and showed variability among clades.

**Supplementary Information:**

The online version contains supplementary material available at 10.1186/s12915-022-01412-1.

## Background

*Candida glabrata* is an opportunistic human yeast pathogen of growing medical concern [1]. Despite its genus name, *C. glabrata* is only distantly related to *Candida albicans,* and belongs to the Nakaseomyces clade, which is more closely related to the non-pathogenic yeast *Saccharomyces cerevisiae* [2]. While the infection mechanisms of *C. glabrata* are not fully understood, several virulence factors have been identified. These include biofilm formation and adherence to host cells mediated by glycosylphosphatidylinositol (GPI)-anchored cell wall adhesins [3], the presence of GPI-anchored proteases [4], or mechanisms to survive the immune system [5]. The availability of a reference genome has enabled comparative genomics approaches to aid in the identification of virulence factors [2]. These analyses uncovered adhesin gene family expansions as the most drastic genomic change underlying the appearance of pathogenicity in the Nakaseomyces clade. Adhesins are large modular proteins whose precursors comprise a secretion signal, a characteristic N-terminal ligand-binding domain, a low-complexity C-terminal domain, and a GPI anchor signal. About two-thirds of the adhesin genes in *C. glabrata* are encoded within subtelomeric regions, and the cell wall can vary in adhesin content depending on environmental conditions or genetic background [6, 7].

The first population genomics analysis in *C. glabrata* identified seven genetically divergent clades [8] across 33 global isolates, and uncovered several gross genome rearrangements and strain-specific gene duplications and losses, with most variability affecting subtelomeric genes encoding adhesins. A more recent analysis of 47 strains consistently found frequent copy number variations in subtelomeric genes [9]. However, both analyses were based on short-read assemblies or short-read mapping to a single reference genome, which limits analysis in several ways. For instance, read mapping only shows variation in genes present in the reference, and results are reference-dependent [10], while short-read assemblies tend to be fragmented and incomplete. These issues can severely impact various downstream analyses, such as gene annotation and pan-genome inference [11–13], and underscore the need for chromosome-level assemblies*.*

Recent developments in long-read sequencing technologies have increased the availability of chromosome-level genomes [14–16], also for *C. glabrata* [17–20]. However, most of these new genomes were highly similar to the reference, as shown by their shared sequence type and conserved structure. To obtain a more diverse dataset of chromosome-level assemblies that fairly represents the known genetic diversity of *C. glabrata*, we used long-read sequencing, available Illumina short reads, and a novel hybrid assembly pipeline to obtain chromosome-level assemblies of twelve additional strains belonging to previously described clades [8]. The final expanded dataset, comprising 21 chromosome-level genomes, allowed us to establish the pan-genome of this important pathogenic species, including a full catalogue of cell wall adhesins. In addition, we reconstructed the evolutionary history of major genomic rearrangements and of variations in the cell wall adhesin repertoire. Our results uncover the dynamics of genome evolution in *C. glabrata* and clarify long-standing questions regarding the actual level of genomic plasticity in this important opportunistic pathogen.

## Results and discussion

### Contiguous assemblies from diverse clades indicate few ancestral large-scale genomic rearrangements

To accurately reconstruct the history of gene content variation and genomic rearrangements in *C. glabrata*, we performed long-read sequencing of twelve strains from clades encompassing the genetic diversity in this species [8]. Combining long reads with available short-read Illumina sequences with the LongHam pipeline (see “Methods”), we obtained highly contiguous hybrid assemblies, consisting in all cases of a single scaffold per chromosome and with most (87%) of the chromosomes including both telomeric regions. Our set includes the reference strain (CBS138), and we compared its assembly to the most recently published one [17]. The two assemblies are highly similar (Additional file 1: Fig. S1), with most differences affecting telomeric regions where some regions were collapsed, and other repetitive regions affected by inefficient correction with Illumina data due to ambiguous mapping. In addition, we used nine available long-read assemblies [19, 20]. Thus, our analysis includes a total of 21 highly contiguous hybrid assemblies, spanning the broad genetic diversity of *C. glabrata* (Table 1).

**Table 1:** Genome statistics

Strain	Source	Genome size (Mb)	Number of contigs	N50	Clade
ATCC90030 (Cg16)	Arastehfar et al	12.51	17	1118927	IVb
B1012M	This study	12.71	13	1061488	I
BG2	This study	12.62	13	1055288	VII
CAS08-0016	Arastehfar et al	12.64	19	1133933	VIb
CAS80027	Arastehfar et al	12.51	16	1057273	V
CAS08-0425	Arastehfar et al	12.35	17	1111896	IVb
CBS138	This study	12.65	13	1141428	V
CST110	This study	12.60	13	1054769	VII
CST35	This study	12.68	13	1073279	IIa
DPK305	Arastehfar et al	12.52	17	1122303	IVb
DPK762	Arastehfar et al	12.57	17	1108465	V
DPL245	Arastehfar et al	12.44	18	1050645	VIb
DSY562	Vale-Silva et al.	12.73	19	1178610	III
DSY565	Vale-Silva et al.	12.73	24	1064559	III
EB0911Sto	This study	12.70	13	1064636	IIa
EF1237Blo1	This study	12.62	13	1133479	IVa
M12	This study	12.62	13	1228727	III
M6	This study	12.61	13	1057064	VII
M7	This study	12.65	13	1067385	I
P35-2	This study	12.69	13	1059622	VIa
SAT_BAL01	This study	12.67	13	1064626	IIb

Legend: Characteristics of the 21 long-read assemblies used in this study. Column 1 indicates strain name, column 2 in which study it has been sequenced, column 3 indicates the genome size in Mb, column 4 indicates number of chromosomes, column 6 indicates the N50 value, and column 7 indicates which clade the strain belongs to

**Fig. 1:**
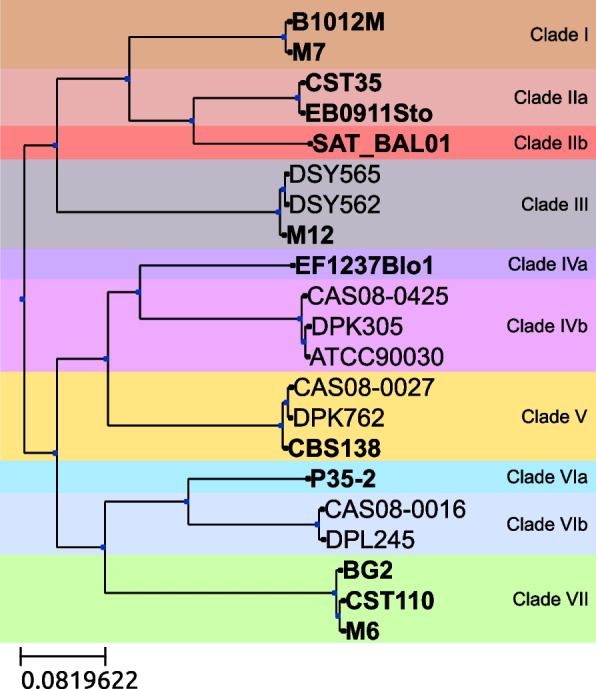
Phylogenetic tree showing the evolutionary relationships of the 21 strains with long-read assemblies. Tree reconstruction was based on a concatenated alignment of 4998 single-copy genes. Identical positions were omitted from the alignment providing an alignment of 105,591 nucleotide positions. A maximum likelihood approach was used to reconstruct the tree and a rapid bootstrap was calculated. All nodes had full support. Strains sequenced in this study are marked in bold. Strains are sorted into clades based on differences between branch lengths leading to the clades and internal branch lengths (see “Methods”)

A previous study [8], including the twelve strains sequenced here, defined seven *C. glabrata* clades based on genome-wide polymorphisms. To classify the remaining nine strains, we reconstructed a phylogenetic tree based on the concatenation of conserved genes (Fig. 1, see “Methods”). Two new strains were added to clades III, V, and VI while three strains were added to clade IV. Our final dataset includes two to four strains per each of the previously defined clades. Based on the presence of long internal branches, we subdivided three of the clades (Fig. 1).

**Fig. 2:**
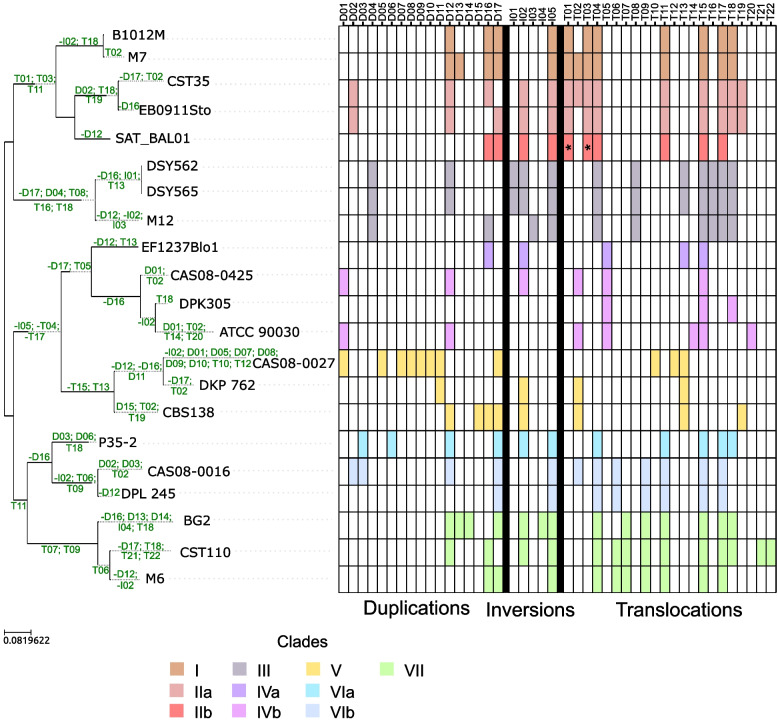
Graph representing structural variation in *C. glabrata* strains. On the left, a phylogenetic tree of the 21 strains is shown. Indicated on the branches are the structural variation events that have occurred at each branch based on parsimony (see events on the heatmap of the right). Events with a - in front were detected in the opposite set of strains but inferred to have happened at the indicated branch based on parsimony. On the right, heatmap summarizing rearrangements found in the 21 strains of *C. glabrata* based on SyRi. The *x*-axis contains a list of rearrangements divided in duplications (D), inversions (I), and translocations (T). The *y*-axis contains the different strains, sorted by clade. Rearrangements are detected by mapping the strain genome against the reference *C. glabrata* genome and using Nucmer and SyRi. White squares mean that the rearrangement is not present in the strain whereas coloured squares mean the rearrangement is present. Squares are coloured according to the clade the strain belongs to and names of the different clades are placed on the upper part of the graph. Squares with an asterisk indicate rearrangements detected as duplications by SyRi but reassigned to other events based on phylogenetic consistency

Structural variation between strains can result in changes in gene expression and can contribute to reproductive isolation [21, 22]. We compared all assemblies to the Sanger-based reference assembly and identified rearrangements (see “Methods”). Circos plots showed some general trends (Additional file 1: Fig. S2), which we explored in more detail using SyRi [23]. On average, strains had eleven large-scale rearrangements (>5000 bp), including duplications, inversions, and translocations (Fig. 2). BG2 was the strain most different from the reference with 19 rearrangements. A similar conclusion was obtained by Muller et al. [24] based on the comparison of the karyotypes of 183 strains of *C. glabrata.* Genomic hybridization results from that study are also congruent with some of the largest translocations detected here: T04 representing the ChrD to ChrI translocation, T05 and T15 representing reciprocal ChrD to ChrL translocations, and T11 and T17 representing the ChrL to ChrI reciprocal translocation. A comparison between *S. cerevisiae* and *C. glabrata* gene order showed that the detected translocations in BG2 were also present in *S. cerevisiae* indicating that these were also present in the *C. glabrata* ancestor.

When comparing general rearrangement patterns between strains, we observed that two of the genomes had unusual patterns. The first, CAS08-0016, belongs to clade VI but did not share any of the large translocations that are found in most of the clades outside clades IV and V (Additional file 1: Fig. S2). The second, CAS08-0425, unexpectedly, was the only clade IVb strain with no rearrangement with respect to the reference (Clade V). To discard assembly artifacts, we re-assembled all genomes from that study [20] using our pipeline. This solved the issues mentioned above (see Additional file 1: Fig. S3) and improved the contiguity of all assemblies except for CAS08-0027 (see genome statistics in Additional file 2: Table S1). The improved assemblies were used in subsequent structural variation analyses.

We grouped rearrangements from different strains based on the rearrangement type, and the reference chromosome region involved, resulting in 17 duplications, 5 inversions, and 22 translocations (Fig. 2 and Additional file 2: Table S2). Thirty-nine percent (17 out of 44) of these rearrangements were strain-specific, most frequent being duplications (9), followed by translocations (6) and inversions (2). We found four clade-specific events, three in clade III (a duplication and two translocations) and one in clade VII (a translocation). We then mapped rearrangements onto the strain tree and used a maximum parsimony approach to infer their evolutionary history (Fig. 2). From 82 events, 49 were inferred to occur at terminal branches of the tree (i.e., very recent, strain-specific events). Events shared by different strains are inferred to have occurred at internal branches with the one subtending clade III being the one with the largest number of rearrangements. We observed a strong tendency for duplications to occur at terminal branches (25 events), as opposed to internal ones (10 events). Such differences were not found among inversions (5 internal and 5 terminal) or translocations (21 internal and 19 terminal). Of note, strains of clades I and II underwent the least number of events, with SAT_BAL01 retaining the genome structure most similar to the inferred *C. glabrata* ancestor. For fourteen of the events (32%), the maximum parsimony scenario inferred multiple gain events.

We compared our results, for the shared strains, to a previous study based on short-read analysis [8]. Of the 69 rearrangements detected in the previous study, 32 (46%) were identified here in the same way (same type of rearrangement and similar coordinates). For the remaining, twelve were considered reciprocal based on short-read data whereas they were in fact unidirectional, twelve indicated different inversions on chromosome L while our data indicate these were a single event, four translocations had wrongly inferred destinations in the short-read study, and the remaining nine events detected based on short reads were non-existent in the long-read assembly and likely correspond to artifacts. Additionally, there were 51 (only translocations and inversions) rearrangements detected with long-read assemblies that were not detected in the short-read based analysis. Thus, comparatively, short reads detect only a subset (in this case 60/111) of the rearrangements detected by long reads, and a fraction of the detected ones (28/60) were interpreted incorrectly in terms of type of events or their placement. Only nine events seem to be artifactual in the short-read dataset, which suggests a low false positive rate, but this approach is characterized by low sensitivity, and low accuracy in terms of defining rearrangement details. These results are in line with the observation that long-read assemblies improve structural variant detection [25, 26]. In addition, the variability in terms of genomic structure observed in *C. glabrata* strains is in line with previous studies [27]. Analyses of karyotypes in *C. glabrata* have shown great variability in chromosome numbers and chromosome sizes, which is also observed in our analysis. For instance, chromosome F ranges from 694 kb in strain CST110 to 1596 kb in strains of clade III due to different translocations. Still, none of our strains seem to contain small chromosomes which can result from duplications of existing chromosomes or translocations of a chromosome arm [28, 29]. Changes in karyotype have been related to different susceptibilities to compounds liable to disturb the fungal cell wall [30] as such we could expect that the different strains presented here could have different reactions to such compounds.

Few studies have been performed that contain multiple genomes assembled using long-read technologies and most of them have been done in human [31]. Here we show how the use of long reads has allowed us to improve the assemblies of twelve strains, as has also been shown before in other genome assemblies [14–18], obtain a broad representation of chromosomal structure across *C. glabrata* diversity, and reconstruct the evolutionary history of major rearrangements. Although most large-scale rearrangements can be detected using short reads, some were incorrectly predicted or became apparent only after the analysis of long-read-based assemblies. Of note, some large-scale rearrangements span multiple clades, implying that the ancestral *C. glabrata* genome structure likely resembles more that of strains in clades I, II, III, VI, or VII than in clades IV or V, which includes the current reference.

### C. glabrata has an open but relatively stable pan-genome

**Fig. 3:**
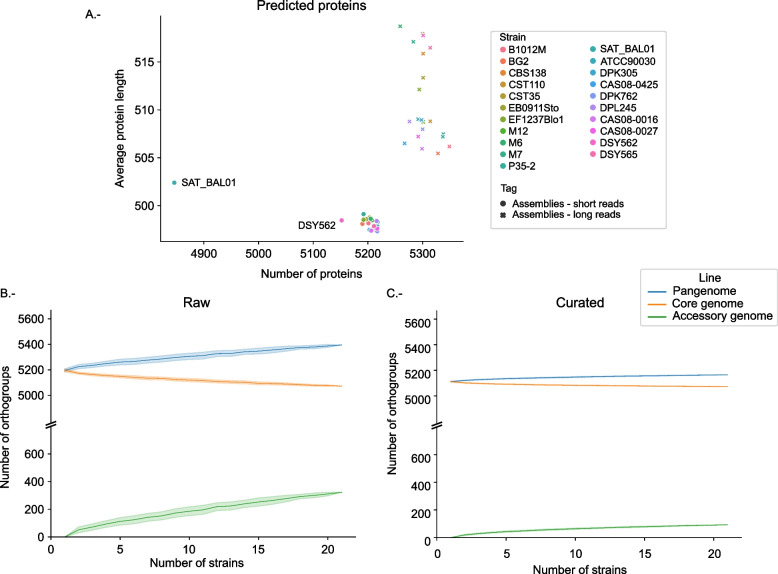
Pan-genome statistics. **A** Scatter plot showing the number of proteins and average protein length in short- and long-read assemblies. **B**,** C** Progression of sizes of the pan-genome (blue), core genome (orange), and accessory (green) genomes with an increasing number of strains. To build the graph, we randomly grouped strains in groups of increasing sizes, from one to the maximum number of strains (21) and for that subset of strains we calculated the size of the pan-genome, core genome, and accessory genome. This was repeated 100 times for each group size, and for each size the average number of proteins in the pan-genome, core genome, and accessory genome were calculated. Standard deviation is shown as a shadow surrounding each line. **B** Build with all orthogroups predicted with orthofinder. **C** Build with all core groups and curated accessory groups. Accessory groups related to miss-annotations or miss-predictions were excluded from the analysis

We next focused on defining *C. glabrata*’s pan-genome. To enable consistent comparisons, we annotated the 21 assemblies using the same gene annotation pipeline, which combines multiple algorithms and incorporates a correction step (see “Methods”). The number of predicted genes per strain ranged between 5259 and 5349 (5302 on average, Additional file 2: Table S3). When compared to the 5280 annotated genes in the Sanger-based reference assembly, most genomes recovered between 97.3% and 98.7% of the genes annotated in the reference genome. In contrast, Illumina-based assemblies for these strains contained 5187 genes on average, which is 2% less genes than in long-read-based assemblies. This difference increases when Illumina coverage is low. For instance, for SAT_BAL01, which has low coverage (92× compared to the average of 329× for all strains), our pipeline predicts 9.2% fewer genes in the Illumina assembly compared to the long-read assembly (4846 vs 5337 genes). Average protein length is also affected in short-read assemblies with an average protein length of 498 amino acids (aa) versus 511 aa in long-read assemblies (Fig. 3A).

Based on the long-read assembly annotations, a total of 5395 orthogroups were defined by orthofinder [32], of which 5072 (94.0%) were present in all 21 strains (core genome), 158 (2.9%) were present in a single strain, and 165 (3,1%) had a variable presence, ranging from two (14 orthogroups) to twenty (82 orthogroups) strains (Additional file 2: Table S4). These results differ from those obtained by a previous study based on short-read assemblies [8] where for a set of 29 strains they established a core genome of only 3603 gene families. A closer examination revealed that such reduced core genome resulted from the inclusion of several highly fragmented assemblies. Note that when the authors took a relaxed core the results were closer to the ones presented here.

To assess whether *C. glabrata* has an open or closed pan-genome, we explored how the pan-genome varied in subsets of randomly chosen individual strains.

Our results (Fig. 3B) show gradual changes in the size of the core and accessory genomes that do not reach a plateau. This suggests that *C. glabrata* has an open pan-genome but also a small accessory genome, and with limited variation in gene content across strains. Despite the use of contiguous assemblies and a consistent gene annotation approach, these inferences can be affected by errors in the assembly sequence and miss-predictions. To assess the impact of assembly or annotation artifacts in pan-genome estimations, we manually curated all 323 accessory families by inspecting annotations, assemblies, and mapping data. Our results (Additional file 1: Fig. S4) indicate that almost 50% of the accessory genome (153 orthogroups) is likely the result of assembly sequence artifacts, as evidenced by discrepancies with the read mapping. Often, unsupported single-nucleotide deletions resulted in frameshifts splitting gene models assigned to different orthogroups. This mostly affected strains with low Illumina coverage. Low coverage in long reads had a different effect, resulting in assemblies missing some gene containing fragments. Annotation problems were infrequent, affecting only 29 (9%) accessory orthogroups.

### Gene fissions, transposable elements, and changes in cell membrane and cell wall repertoires underlie most gene content variations in C. glabrata

After manual curation, 93 (29%) accessory orthogroups were confirmed, of which 46 were present in five or fewer strains: four are de novo gene formations and 42 are the result of bona fide gene fissions. Gene fissions often led to the presence of two proteins, one of which (the longest) was sometimes assigned to the orthogroup of the ancestral gene, with the other one forming an accessory family. Accessory genes were enriched in Gene Ontology (GO) terms related to cell wall and membranes (GO0005199 - Structural constituent of cell wall, GO0005618 - Cell wall, GO0005886 - plasma membrane, GO0005938 - cell cortex, GO0016020 - membrane) and with glycosylation (GO:0016757 - transferase activity, transferring glycosyl groups, GO:0006486 - protein glycosylation). Importantly, none of the duplicate pairs (ohnologs) resulting from the ancestral yeast allopolyploidization [33, 34] was found to be among the accessory gene repertoire. We also identified 107 orthogroups that had been duplicated in at least one of the strains and found that these were enriched in terms such as transmembrane transport (GO:0055085), cell wall (GO:0005618), and integral component of membrane (GO:0016021).

We next searched for accessory genes that were either present or absent in a single clade, as phylogenetic consistency provides further support for the existence of a true gene. There were 23 such orthogroups, of which 17 were clade-specific and six were lost specifically in one clade. One orthogroup emerged through de novo gene formation in clade V. This gene (CAGL0L04836g) was found in all three strains of clade V, encodes a 65 aa protein of unknown function, and emerged through a mutation that changed a stop codon into a glutamine codon. This gene does not have homologs in other, closely related, species according to YGOB [35]. Another accessory orthogroup originated through gene conversion. The *TIR1* gene in *S. cerevisiae* encodes a core cell wall protein and has several paralogs in *C. glabrata*. In CBS138, two identical *TIR* paralogs (CAGL0H09592g and CAGL0H09614g) are adjacent and form part of the core orthogroup OG0000046, which is duplicated only in clade V strains. Orthogroup OG0005179 also contains paralogs of this gene that are grouped separately because they are shorter due to a 96-bp deletion. The most likely scenario is that *TIR1* was duplicated in the *C. glabrata* ancestor and shortly thereafter one of the copies acquired the deletion. Then, a case of gene conversion restored the two original copies in the ancestor of clade V.

Among the other accessory orthogroups, represented in more strains, we find three for which read mapping indicates that the whole gene fragment is missing in strains lacking the gene. Unlike previous examples, where small mutations caused the fragmentation of a gene, these cases represent DNA acquisition. A closer look revealed they were transposable elements (TEs). *C. glabrata* encodes few TEs, with the reference genome encoding only Ty3 elements [36] and with Ty5 elements having been recently identified in three strains [18, 19]. We identified Ty3 homologs in strains from clade V and clade VII (see OG0005204 in Additional file 2: Table S4). Interestingly, given the conservation so far observed among *C. glabrata* strains, members of clade VII had the transposon inserted in chromosome A. Clade Va was more diverse. The three clade Va members had one Ty3 transposon inserted in chromosome G, but then DPK762 had an additional copy in chromosome M and CAS08-0027 had five additional copies in chromosomes A, D, G, I, K, and M. These differences in chromosome location suggest active transposition of Ty3. We also found several Ty5 homologs beyond the ones described previously [18, 19], with strains from the same clade having variable numbers and locations of TEs (see OG0000043 in Additional file 2: Table S4). Vale-Silva found nine copies of Ty5 in DSY562 and eight in DSY565. They divided the copies in two subgroups based on similarity, the first included TPK5-1, TPK5-3, and TPK5-4 (Ty5A) whereas the second included TPK5-2 and TPK5-5 to TPK5-9 (Ty5B). Xu et al. [18] found one copy of Ty5B in BG2 a truncated copy that belonged to Ty5A. In our dataset, M12, from the same clade as DSY562 (clade III), has seven copies, two less than DSY562. Based on synteny, it appears to lack TPK5-1 and TPK5-8, but it contains TPK5-7, which is absent from DSY565. Our results for BG2 are congruent with the results from Xu et al. We analyzed the two other members of clade VII (CST110 and M6) for the presence of TEs. Neither of them had the full copy of the Ty5B found in BG2 but they contained the truncated copy in Ty5A. Further copies of Ty5 were also found in members of clade IIa and VIa. CST35 (clade IIa) has a copy of TPK5-1 and a copy of either TPK5-3 or TPK5-4, both in chromosome J. EB0911Sto, from the same clade, also has a copy of TPK5-1 but the other copy appears to be truncated. Finally P35-2 from clade VIa contained a truncated copy of the Ty5B in chromosome A and a copy of Ty5A in chromosome J. These findings are evidence of a dynamic activity of TEs in the recent evolution of *C. glabrata*.

### A complete catalogue of adhesins and assessment of their variability across strains

Adherence, mediated by adhesins, is a crucial phenotype for *C. glabrata* pathogenesis [37]. Adhesins have been shown to vary in terms of copy numbers across *C. glabrata* strains [8]. However, adhesins are often encoded in subtelomeric regions and are therefore often missing from short-read assemblies, which complicates establishing the complete adhesin repertoire for the species, as well as tracing intra-specific changes. To fill this gap, we developed a specific computational pipeline (see “Methods,” Additional file 1: Fig. S5) to search for adhesin-coding genes in the 21 long-read based assemblies. We predicted a total of 1548 adhesins in the 21 strains. We compared the results obtained by the same pipeline on our Illumina-based assemblies and saw that, as expected, on average more than 10% of the predicted adhesins were missing. We grouped the 1548 adhesins into 83 adhesin orthogroups (see “Methods”) and found that four (named New Adhesin Orthogroups, NAO) were absent from the two strains for which curated adhesin datasets exist (CBS138, BG2) [17, 18]. Thus, we expanded the catalogue of known adhesins by 4.8%. On average, each strain encodes 74 adhesins, ranging from 67 in CAS08-0425 to 81 in EB0911Sto. DSY562 and DSY565 previously have been reported to have 101 and 107 adhesins, respectively [19] whereas based on our pipeline and manual curation both have 79, though not exactly the same set.

**Fig. 4:**
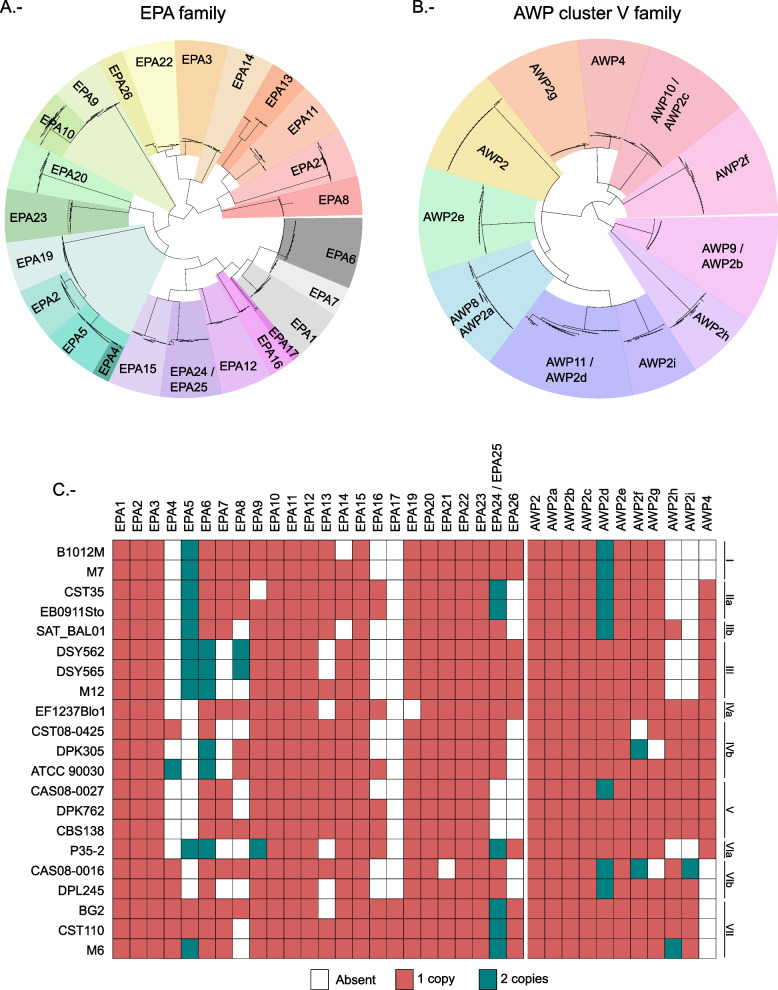
Adhesin evolution. **A** Phylogenetic tree reconstructed for the EPA family. **B** Phylogenetic tree reconstructed for the AWP2 cluster V family containing the 11 closest related orthogroups. Both trees are based on the first 900 nucleotides of each gene. **C** Heatmap showing the absence, presence, and duplication pattern for each gene included in the trees and for each strain

**Fig. 5:**
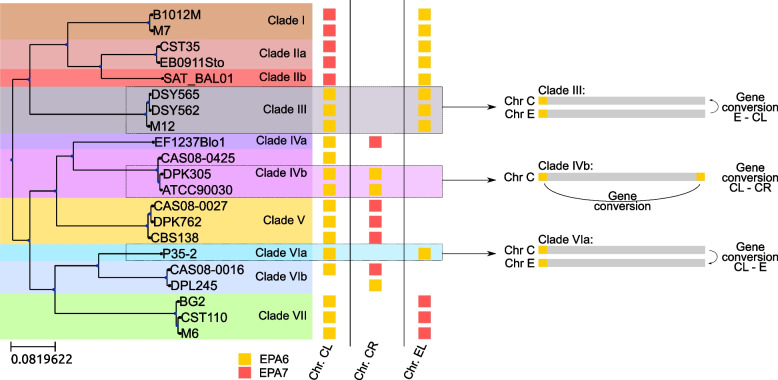
Graph representing the putative gene conversion events that have happened between *EPA6* and *EPA7*. Shown on the left is the species tree of the 21 strains of *C. glabrata*, followed by a schematic representation of the presence of *EPA6* (yellow squares) and *EPA7* (red squares) in the three loci: left arm of chromosome C (CL), right arm of chromosome C (CR), and left arm of chromosome E (EL). Shown on the right is a schematic representation of the gene conversion events

Seventy adhesin orthogroups per strain were identified on average, ranging from 65 in DPL245 and CAS08-0425 to 73 in EB0911Sto and CBS138. Seventeen of the adhesin orthogroups comprised paralogous genes in at least one of the strains. Using parsimony, we estimated that the last common ancestor of *C. glabrata* encoded 81 of the 83 adhesin orthogroups. The remaining two adhesin orthogroups were inferred to have been acquired through duplication at different nodes in the *C. glabrata* phylogeny (Additional file 1: Fig. S6). *EPA17* was present exclusively in clade VII where it emerged through duplication, and is closely related to *EPA1*, *EPA6*, and *EPA7*, all of which have been related to biofilm formation [38]. The other adhesin is one of the new adhesin orthogroups (NAO_4) which emerged before the divergence of clades I, IIa, and IIb, and then was lost in clade IIb. This adhesin orthogroup is closely related to CAGL0J00132g, which was recently described as adhesin-like [17]. A closer look into the evolutionary events that have shaped some of the main adhesin families, EPA and AWP2 cluster V (see Fig. 4 and Supplementary text) revealed at least three pairs of adhesin orthogroups affected by gene conversion with the clearest case found between the *EPA6* / *EPA7* pair (see Fig. 5). Duplication events or gene losses have also changed the adhesin content of several strains. For instance, *AWP2d* is duplicated in several strains, and a closer analysis showed that the duplication was ancestral and followed by differential gene loss (see Additional file 3 for more details) [7, 20, 39, 40].

Unlike most *C. glabrata* strains, basal adhesion of DSY strains (DSY562 and DSY565) to epithelial mammalian cell lines does not seem to be mediated by Epa1 [41]. Deletion of *EPA1* in these hyper adhesive strains reduces adhesion to basal levels observed in other strains indicating that in these strains adhesion is not only mediated by *EPA1*. In an effort to identify other adhesins involved in adhesion of these strains, the content of adhesins for DSY strains was compared to the one in the CBS138 reference. However, between CBS138 and the DSY strains, there are 12 adhesins missing and five had a duplication in one of the strains but not the other. This made it difficult to hypothesize which adhesin was responsible for the adhesion in the EPA1 knockout. As seen in our results, clade V strain CBS138 is far related from DSY562. Strain M12, on the other hand, belongs to the same clade and as such is a better candidate to compare adhesin content. Between strains of clade III, there are 3 missing adhesins and four of them have a duplication only in one strain. Additionally, M12 has not been shown to be hyper-adherent [8] though whether it is dependent on Epa1 for adhesion is still an open question. It is likely, however, that M12 does not share the same adherence mechanism as DSY strains. The difference in adhesin content between these closely related strains is as follows: duplications in DSY strains involving the repetitive region found in chromosome C (involving genes CAGL0C00803g, CAGL0C00825g, and CAGL0C01133g), the duplication of *EPA8*, which is completely missing in M12, the loss of *EPA13* in DSY strains while it is present in M12, a duplication of *PWP4* found specifically in M12, and the presence of a gene of one of the new adhesin orthogroups in DSY562 (NAO1). Given the known role of Epa proteins in adhesion, we hypothesize that the duplications of *EPA8* could be related to the basal adhesion found in DSY strains when *EPA1* is not functional.

## Conclusions

The use of chromosome-level assemblies of strains representing the breadth of *C. glabrata* genetic diversity has allowed us to obtain an accurate and comprehensive picture of the evolution of the genomic architecture, the genetic content, and the repertoire of adhesion proteins in this important yeast pathogen. Contrary to the general assumption of a highly plastic genome for this species, and considering the large evolutionary distances involved, we found relatively few large-scale rearrangements and limited gene content variations. We argue that the high genome plasticity assumption is a delusion in part caused by observations made from comparisons unaware of the phylogenetic context (i.e., the large evolutionary distance between the compared strains), and the many artifactual differences that can result from the use of short-read assemblies, as shown in this study. This situation can be the case for other eukaryotic species for which the number of long-read assemblies is still limited. We found that gene content variation mostly affects genes encoding cell wall proteins, and that fission, duplication, or loss of pre-existing genes, rather than de novo formation or horizontal acquisition of genes are the main forces driving pan-genome evolution in *C. glabrata*. Still, the rather small dataset and the limited variation found in the *C. glabrata* pan-genome show that such events are sparse and of limited effect on the evolution of *C. glabrata*. Our results also provide evidence of recent activity of the few transposon families present in this yeast. Our detailed analysis of the adhesin repertoire uncovered four previously unknown adhesin orthogroups and thus expanded the catalogue of these relevant proteins. The adhesin core genome contains 44 genes representing 53% of the complete adhesin set, as compared to 94% overall gene content in the core genome. Each clade or strain presents a subset of 60 to 73 adhesin orthogroups drawn from a total species catalogue of 83, most of which were already present in the common ancestor of *C. glabrata*. Of note, none of the strains analyzed here contained the exact same set of adhesins. Gene loss, duplication, and conversion drive the evolution of these in majority subtelomeric genes. Thus, *C. glabrata* entails a plastic adhesin repertoire within an overall mostly static pan-genome, reminiscent of the two-speed genome hypothesis put forward for filamentous plant pathogens [42]. This variability in adhesin repertoires could underlie variation in adhesion properties of each of the different strains.

## Methods

### Genome sequencing

For PacBio sequencing (strains CST35, M6, and M7), genomic DNA was extracted from *C. glabrata* isolates using a MasterPure™ Yeast DNA Purification Kit (Epicentre) with slight modifications. Since a considerable amount of gDNA was needed, the extractions were performed from multiple replicates that were pooled into one final sample preparation. In brief, strains were grown overnight in liquid YPD at 37°C, after which the cells were pelleted and lysed with RNAse treatment at 65°C for 15 min. Samples were cooled on ice for 5 min and treated with purifying Protein Precipitation kit reagent by vortexing, centrifugation, and transferring the supernatant to a new Eppendorf tube as described in the kit protocol. After that, the genomic DNA was precipitated with cold absolute ethanol (samples were left at −20°C with the ethanol for at least 2 h) and then was pelleted by centrifugation at 16,000*g* for 30 min at 4°C. The pellet was washed in 70% ethanol, left to dry and resuspended in TE buffer. The Genomic DNA Clean & Concentrator kit (Zymo Research) was used for the final purification. Samples were then sent to the Functional Genomics Center at University of Zurich/ETH Zurich for PacBio sequencing. The SMRT bell was produced using the DNA Template Prep Kit 1.0 (Pacific Biosciences). The input genomic DNA concentration was measured using a Qubit Fluorometer dsDNA Broad Range assay (Thermo Fisher). Five micrograms of gDNA was mechanically sheared to an average size distribution of 15–20 kb, using a gTube (Covaris). A Bioanalyzer 2100 12K DNA Chip assay (Agilent) was used to assess the fragment size distribution. Five micrograms of sheared gDNA was DNA damage repaired and end-repaired using polishing enzymes. A blunt end ligation reaction followed by exonuclease treatment was performed to create the SMRT bell template. A Blue Pippin device (Sage Science) was used to size select the SMRT bell template and enrich the big fragments >8 kb. The size-selected library was quality inspected and quantified on the Agilent Bioanalyzer 12-kb DNA Chip and on a Qubit Fluorimeter (Thermo Fisher), respectively. A ready-to-sequence SMRT bell-Polymerase Complex was created using the P6 DNA/Polymerase binding kit 2.0 (Pacific Biosciences) according to the manufacturer’s instructions. The Pacific Biosciences RS2 instrument was programmed to load and sequence the sample on one SMRT cell v3.0 (Pacific Biosciences), taking a movie of 240 min per SMRT cell. A MagBead loading (Pacific Biosciences) method was chosen to improve the enrichment of the longer fragments. After the run, a sequencing report was generated for each cell, via the SMRT portal, in order to assess the adapter dimer contamination, the sample loading efficiency, the obtained average read-length, and the number of filtered sub-reads.

For Nanopore sequencing (strains B1012M, BG2, CST110, CBS138, EB0911Sto, EF1237Blo1, M12, P35-2, and SAT_BAL01), genomic DNA was isolated similarly as for PacBio with few modifications. Here, we performed the RNAse treatment twice. Once following the protocol and with the RNAse provided with the kit for 15 min at 65°C, and, secondly, on the supernatant after the treatment with the Protein Precipitation reagent—3 μL RNAse T1+A, 37°C for 30 min. We also performed phenol-chloroform purification using PLG Heavy tubes. After that, the samples were left at −20°C with 3 M sodium acetate, cold absolute ethanol, and glycogen for at least 2h for genomic DNA precipitation. The DNA was pelleted by centrifugation at 16,000*g* for 30 min at 4°C and washed in 70% ethanol, left to dry, and resuspended in TE buffer.

Nanopore sequencing was performed at the Ultra-sequencing core facility of the Centre Nacional d’Analisi Genòmica (CNAG) in Barcelona (Spain). Integrity of genomic DNA was analyzed by pulse field electrophoresis (Pippin Pulse, Sage Science), and contamination of DNA samples was checked with NanoDrop (Thermo Fisher Scientific) spectrophotometry based on 260/280 and 260/230 ratios. Then, the samples were used to prepare ten 1D2 and 1D genomic libraries using the Ligation sequencing kits SQK-LSK308 and SQK-LSK108 for sequencing on a MinION instrument (Oxford Nanopore Technologies, ONT). Two micrograms of genomic DNA was nick-repaired using the NEBNext FFPE DNA Repair Mix (NEB, M6630) and purified with 0.4× Agencourt AMPure XP Beads (Beckman Coulter, A63882). Samples were end-repaired and dA-tailed using the NEBNext UltraII End Repair/dA-Tailing Module (NEB, E7546) and subsequently purified with 1× Agencourt AMPure XP Beads. When using the SQK-LSK108 kit, the 1D sequencing Adapter Mix (AMX1D, ONT) was ligated to the purified samples using the Blunt/TA Ligase Master Mix (NEB, M0367L). When using the SQK-LSK308 kit, two adapter ligation steps were required. First, the 1D2 adapter was ligated to the purified samples using the Blunt/TA Ligase Master Mix. After a 0.4× purification with Agencourt AMPure XP Beads, the product with the 1D2 adapter was ligated to the BAM sequencing adapter (ONT) using the Blunt/TA Ligase Master Mix. The adapter-ligated products from both kit versions were purified using 0.4-fold excess of AMPure XP beads. The beads were washed twice using the Adapter Bead Binding Buffer (ABB, ONT), and the libraries were eluted in 15 μl of Elution Buffer (ELB, ONT).

Libraries prepared with the SQK-LSK308 kit were loaded into R9.5 or R9.5.1 chemistry FLO-MIN107 flow cells (ONT), and libraries prepared with the SQK-LSK108 kit were loaded into R9.4 chemistry FLO-MIN106 flow cells (ONT) according to manufacturer’s recommendations. In brief, first, the MinKNOW interface QC (ONT) was run to assess the flow cell quality, and this was followed by the flow cell priming. The sequencing library was mixed with a running buffer, Library Loading Beads (ONT), and nuclease-free water and loaded onto a “spot on” port for sequencing. Sequencing data was collected during 48 h. The quality parameters of the sequencing runs were further monitored by the MinKNOW platform while the run was base-called using the Albacore 2.3.1.

### Public assemblies and sequencing reads

Nine published long-read-based assemblies were included in the analysis. Seven strains were published by Arastehfar et al. [20], and the additional two strains were published by Vale-Silva et al. [19] (see Table 1). Genomes were downloaded from NCBI (DSY562 and DSY565 from PRJNA374542, and the remaining seven from PRJNA718446). The CBS138 assembly from Xu et al. [17], used for comparative purposes, was downloaded from PRJNA596126. Illumina reads used for the assemblies of the twelve strains sequenced here were downloaded from PRJNA361477, PRJNA506893, and PRJNA635652 [8].

### Genome assemblies

We developed a novel hybrid pipeline (long-read hybrid assembly merger - LongHam: available at github https://github.com/Gabaldonlab/longHam) to obtain the twelve new chromosome-level assemblies. This pipeline uses six different assembly programs to produce five different primary assemblies, which are subsequently combined using Ragout [43]. The steps in the pipeline are detailed next (see also Additional file 1: Fig. S7 for a scheme).

First, Illumina reads were trimmed using Trimmomatic v0.38 [44] (parameters: LEADING:3 TRAILING:3 SLIDINGWINDOW:4:15 MINLEN:36) and long reads were trimmed using porechop v0.2.4 [45] (parameters: default). Nanopore reads were additionally trimmed using nanofilt 2.5.0 [46] (parameters: -q 7). Long reads were then corrected using CANU v1.8 [47] (parameters: default). A subset of corrected long reads that represented a genome coverage of 30× formed by the longest reads was extracted and used as input in the different assembly approaches. Five different primary genome assemblies were built using each of the following approaches: (A) MaSuRCa v3.4.2 [48] (parameters: default); (B) Canu v1.8 [47] (parameters: default); (C) WTDBG2 v2.1 [49] (parameters: default); (D) a combination of Illumina assembly with platanus v1.2.4 [50] (parameters: default) and the DBG2OLC v20180222 [51] (parameters: k 17 AdaptiveTh 0.01 KmerCovTh 10 MinOverlap 100 RemoveChimera 2 ContigTh 2), and (E) a combination of SparseAssembler v20160205 [52] (parameters: LD 0 k 51 g 15 NodeCovTh 1 EdgeCovTh 0) and DBG2OLC v20180222 [51] (parameters: k 17 AdaptiveTh 0.01 KmerCovTh 10 MinOverlap 100 RemoveChimera 2 ContigTh 2). These programs cover a variety of approaches. MaSuRCa (A) is a hybrid assembly approach that combines long- and short-read data. Canu (B) and WTDBG2 (C) use long reads exclusively, with Canu incorporating a correction step that is later used for all other programs in the pipeline. Finally, DBG2OLC is a scaffolder that uses long reads to join fragmented Illumina assemblies. It is used twice; with an Illumina-based assembly built with platanus (D) and with an Illumina-based assembly reconstructed with SparseAssembler (E). Each primary assembly was individually corrected with three iterations of Pilon v1.22 [53] (parameters: default). Then, different combinations of primary assemblies were fused into combined assemblies using Ragout v2.0 [43] (parameters: default). For this, four different assembly combinations were built (Additional file 1: Fig. S7) but the best results, based on the number of contigs and N50, were consistently obtained by scaffolding the Canu assembly (B) with the assemblies built with platanus + DBG2OLC (D) and WTDBG2 (C). This combined assembly was corrected again with Pilon. This procedure resulted in highly contiguous genome assemblies for the twelve strains, albeit with some strains containing a few split chromosomes. Such split chromosomes were corrected by scanning alternative primary assemblies of the same strain for the presence of that particular chromosome in a complete form. This last step allowed us to obtain a full set of chromosomes for all twelve assemblies (see Additional file 2: Table S5 for a list of changes). We also applied LongHam to the seven assemblies obtained from Arastehfar et al. [20] due to inconsistencies observed when analyzing genome rearrangements. These assemblies were not as complete as in the previous set of strains due to shorter Nanopore read lengths. Results from Ragout were not usable, and we composed genomes by picking complete chromosomes from the primary assemblies. This was done for six out of the seven genomes. The 7th was too fragmented in all primary assemblies to establish a reliable chromosome-level assembly (see Additional file 2: Table S1 for genome statistics). These genomes were used exclusively for the rearrangement analysis. All other analyses run in this study were performed on the originally submitted genomes.

Additionally, our assembly for the reference strain CBS138 was scanned for the presence of telomeric repeats, and some of the chromosomes lacking telomeric repeats were replaced by their versions in other primary assemblies that included telomeric repeats, as described for split chromosomes (see Additional file 2: Table S5 for changes). The same process was attempted for the remaining strains, but no good candidates were found. Comparisons between our CBS138 assembly and the one published by Xu et al. [17] showed several differences between the two assemblies. We finally added an additional correction step based on long reads using a combination of minimap2 v2.9-r720 [54] and racon v1.4.14 [55], the assembly improved slightly in terms of similarity with the assembly published by Xu et al. [17]. This final assembly was the one used in the following analyses. The differences observed between our CBS138 and the genome provided by Xu et al. indicates that the genomes provided, despite being chromosome-level assemblies, will still have issues in repetitive regions.

### Genome rearrangements

Strain genomes were aligned to the Sanger reference genome using Nucmer v4.0.0rc1 [56] (parameters: --maxmatch -c 100 -b 500 -l 50). Nucmer mappings were filtered to keep only those with an identity above 98% and a minimum length of 5000 bp. Then, SyRi V1.5 was run to assess genome rearrangements [23] (parameters: --nosnp). In case the strain genome was not assembled at the chromosome level, the chroder application from SyRi was run beforehand. This application joins scaffolds based on the reference genome. As our strain genomes are not very fragmented, this should not have a big impact on the prediction of rearrangements. We manually ensured that the direction of chromosome L was consistent across genomes as the large inversion sometimes caused chroder to place it in the reverse direction. Rearrangements were then grouped into common events based on the reference and strain chromosomes involved and the piece of the reference genome that was involved. Note that duplications 12, 16, and 17 (Fig. 2B) can contain multiple events per strain but were grouped together due to the difficulty of establishing clear, non-overlapping events. Events were then mapped onto the species tree assuming they occurred at nodes where all leaves contained the same event. As such, some events were considered to happen multiple times in parallel. Two duplications in the SAT_BAL01 genome were reassigned to translocation events based on phylogenetic consistency and consistency of the event in the primary assemblies performed during LongHam assembly.

### Genome annotation

Genome annotation was performed on each genome assembly individually using a combination of methods. To allow comparable results, the same pipeline was used to annotate the newly generated assemblies and the publicly available ones. The set of reference proteins to be used for homology-based predictions were formed by the protein-coding genes in the annotation of the *C. glabrata* CBS138 Sanger reference (downloaded in Nov 2018 from https://candidagenome.org) [57], and the collection of 20 proteomes used in YGOB [35]. Exonerate v2.4.0 [58] (parameters: --showtargetgff TRUE -m p2g --showalignment FALSE --showvulgar FALSE -n 5) was used to search and annotate genes coding every protein in YGOB in each of the assemblies. RATT (downloaded in 2018) from the PAGIT v1 package [59] was used to transfer annotations from the Sanger CBS138 annotation to the strain genomes. This was done by running the program in three modes: strain, species, and multi, and the method with the highest number of transferred gene annotations was kept. YGAP web server [60] (accessed October 2020) and MAKER2 v2.31.10 [61] (parameters: default) were used to obtain two additional predicted gene sets. Results from RATT, YGAP, MAKER2, and exonerate were combined into a single gene prediction using EVM (downloaded in 2018) [62] (programs / parameters: partition_EVM_inputs.pl: --segmentSize 100000 --overlapSize 10000; write_EVM_commands.pl with equal weights for all predictions; execute_EVM_commands.pl; recombine_EVM_partial_outputs.pl; convert_EVM_outputs_to_GFF3.pl). This annotation was then improved by specifically searching for the presence of genes that were predicted in the *C. glabrata* reference but not included in the strain annotations. For this, we exploited the fact that *C. glabrata* strains are highly syntenic. First, the pipeline associated each predicted protein in the strain genome to the reference genome using a best reciprocal hit (BRH) approach. Then, unmatched genes were linked to reference genes with enough similarity (>50% identity) based on their genomic location. In the same way, the pipeline corrected spurious BRH matches that were not congruent with the gene order conservation. Then, for each putative missing gene, the pipeline scanned for RATT annotations for those genes. If found, they were incorporated into the gene prediction. If not, the pipeline located surrounding genes and then used GTH v1.7.1 [63] (parameters: -gff3out -species fission_yeast -skipalignmentout) to search the intergenic space between those genes for the potential presence of the missing genes. In a last step, for the remaining missing genes, the whole genome was scanned for their presence. We assessed the similarity between annotations of the different strains and the reference Sanger annotation based on BRH.

### Identification of adhesin genes

We designed a specific pipeline for the detection of adhesin genes. This pipeline first joins all the independent gene annotations made in the gene annotation step (see above), together with all possible open reading frames that are 100 aa long or longer. Additionally, exonerate is used on the genomic sequences inspected to detect the presence of homologs of the 81 adhesins annotated by Xu et al. [17] in the reference strain CBS138. Combined, these annotations form the full potential proteome set for each strain. We then used complementary strategies to identify adhesins among these proteome sets. The first strategy is based on the use of HMMER profiles to scan for putative adhesins. Profiles were built based on the N-terminal regions of the 81 predicted adhesins from Xu et al. [17]. For this, the first 300 aa of each protein were extracted and then a BlastP search was used to search for homologs within the dataset. MCL v14-137 [64] was used to group homologous proteins into families. These families were then used to create a profile using the hmmbuild option of HMMER v3.0 [65] for those groups that contained at least three homologs. Then hmmsearch was used to scan the proteome set for adhesins. Results were filtered with an e-value < 0.1 for the whole prediction and an e-value of < 0.1 for the first domain. The second strategy consisted of blast searches performed using the 300 first amino acids of the proteins of Xu et al. as reference. Finally, SignalP v5 [66] was used to detect whether the predicted proteins contain a signal peptide (SP) for secretion. As the complete proteome dataset is highly redundant, we grouped adhesin candidates according to their location in the genome (i.e., all predicted sequences from a putative single locus), then we selected one representative sequence of each putative adhesin by choosing the longest sequence among those containing a SP (see Additional file 1: Fig. S5). This pipeline was first run for CBS138 [17] and for BG2 [18] strains, for which a set of manually annotated adhesins is available. This allowed us to optimize the parameters of the pipeline and omit some of the HMMER-based families that were producing false positives. Subsequently, we used the pipeline to detect adhesins in the genome assemblies for the remaining 19 strains. In addition, a complementary search for adhesins in the 21 strains was performed focusing on the presence of C-terminal GPI-anchoring peptides or internal adhesin repeats. Proteins larger than 500 aa containing C-terminal GPI-anchoring peptides but lacking internal transmembrane domains (TMHMM - 2.0) were selected as well as proteins (or fragments) containing characteristic adhesin VSHITT and SFFIT repeat motifs, which are a class of tandem repeats found in *C. glabrata* that have been found in adhesins [67, 68]. GPI-anchoring peptides were identified using the Big-Pi Fungal predictor in combination with a complementary pattern + composition scanning approach and ProFASTA parsing [69]. Repeat motif searches were also performed with ProFASTA. Any selected protein not picked up in the previous pipeline was analyzed by Blast to eliminate false positives with homology to non-adhesin proteins. The remaining ones, mostly internal or C-terminal fragments of already identified adhesins, were added to the list of putative adhesins. Finally, the complete list of putative adhesins was manually curated; proteins that did not meet the criteria described above were removed (including some predicted adhesins from Xu et al. [17]), and protein boundaries were checked against published reference sequences (see Additional file 2: Table S6). Note that the inability to retrieve complete adhesin sequences indicates that despite the telomere to telomere assemblies obtained there are likely still problems in highly repetitive regions, where Illumina reads are not able to correct the long reads. Still, this has shown to have a limited impact seeing we are able to retrieve nearly all adhesins predicted by previous studies of CBS138 and BG2.

After identifying all adhesins and their fragments (see Additional file 2: Table S5), we grouped all adhesins into families using MCL v14-137 using only the N-terminal region of each EPA protein (i.e., the first 300 amino acids). Then, for each family we reconstructed a phylogenetic tree using IQTREE v1.6.9 [70] (see Additional file 2: Table S7). Trees were manually scanned and then split into different families when long branches divided the trees into different, well supported, clades (see Additional file 1: Fig. S8). A total of 83 adhesin orthogroups were defined in this way (see Additional file 2: Table S8). The detected adhesins were added to the main gene annotation if they were missing.

### Definition of orthogroups for 21 reference strains

Orthofinder v2.2.7 [32] was used to group annotated protein-coding genes into orthogroups. This was first done for the 21 strains with long-read assemblies. Orthogroups were assigned to the core genome when they had homologs in all strains and to the accessory when they were missing in any of the strains. Gene orthogroups in the accessory genome were manually curated by inspection of read mappings, blast searches, and exonerate searches. Accessory families were divided according to their reliability and the assigned cause for their accessory nature, see Additional file 1: Fig. S9 for a schematic of the different results. Families that were deemed as part of the accessory genome due to indels not supported by read mappings were tagged as sequencing errors, if the reason was a problem in the gene annotation they were labeled as miss-predicted. Some cases were found in repetitive regions where poor Illumina read mapping prevented proper curation, those cases were labeled as unclear. Finally, genuine accessory families could be the result of a mutation or indel that caused a gene fission, a mutation of indel that caused a de novo gene formation or because of the presence of a piece of DNA not present in some strains were considered bona fide accessory families. Additional file 2: Table S3 contains the list of orthogroups forming the *C. glabrata* pan-genome.

### Functional annotation of orthogroups and functional enrichment

We ran interproscan v5 [71] for each strain. Then we checked whether any of the defined orthogroups was enriched using an in-house version of FatiGO [72]. Enrichment was considered with an adjusted *p*-value < 0.01. REVIGO [73] was used to filter enriched GO terms.

### Strain tree reconstruction

Based on results of orthofinder, we concatenated single-copy genes found in the core genome of the set of 21 strains and the complete set of strains. Then we omitted positions that were identical in all strains using trimAl v1.4.rev22 [74] (-st 1 -complementary) and obtained a final alignment spanning 105,591 nucleotide positions. IQTREE v1.6.9 [70] was used to infer the best model and reconstruct the species trees. One thousand rapid bootstraps were calculated. Clades were delimited as follows: first ETE v3 [75] was used to build a matrix of all distances between all strains based on branch lengths. Strains were then grouped using the KMeans metric. All possible *K* values were tested. For all the groups defined in the different KMeans runs, we selected those that were monophyletic and that had the biggest difference between the branch length of the node and its children nodes (see in the tree that clades are identified by a long branch followed by many shorter branches). To make this calculation, we first obtained the node branch length (bl1) and the average of the children branch lengths (bl2). As many of the branch lengths are smaller than 1, we used log10 to normalize both branch lengths and then divided bl2 by bl1. The larger this score is the more difference there is between bl1 and bl2. Then we sorted all groups based on the score and took the largest until all strains were set within a group or remained unplaced. This resulted in the 10 clades presented in this paper.

### Illumina genome assembly and annotation

We assembled the Illumina reads into a short-read based assembly using Spades v3.13.0 [76] (parameters: default). Genes were annotated using the same pipeline as seen above. The only change was the limitation of YGAP to not have more than 702 contigs; therefore, for this analysis, we selected only the 700 longest contigs for each Illumina-based assembly. Adhesins were also calculated using our in-house pipeline but were not manually curated.

## Supplementary Information

**Additional file 1: Fig S1.** Comparison of reference assemblies. **Fig S2.** Circos plots representing structural variation among strains. **Fig S3.** Dotplot representing improvements in assembly of strain CAS08-0016. **Fig S4.** Barplot representing origin of accessory families. **Fig S5.** Graphical representation of the adhesin detection pipeline. **Fig S6.** Gains and losses of adhesin families in the *C. glabrata* strains. **Fig S7.** Summary of the LongHam pipeline. **Fig S8.** Example of an adhesin gene cluster. **Fig S9.** Schematic representation of the manual curation process to validate accessory genes.

**Additional file 2: Table S1.** Assembly statistics for the re-assembly of the genomes from Arastehfar et al. [20]. **Table S2.** List of rearrangements when compared to the *C. glabrata* sanger reference strain. **Table S3.** Summary of the results of the gene annotation for the 21 *C. glabrata* strains. **Table S4.** Complete list of orthogroups as determined by orthofinder. **Table S5.** List of chromosome substitutions during assembly. **Table S6.** List of predicted adhesins for the 21 *C. glabrata* strains. **Table S7.** Phylogenetic trees reconstructed to group adhesins into families. **Table S8.** List of adhesin orthogroups as determined based on phylogenetic analysis.

**Additional file 3.** Chromosome-level assemblies from diverse clades reveal limited structural and gene content variation in the genome of Candida glabrata.

## Data Availability

Genome assemblies and long-read data can be found in NCBI under bioproject PRJNA717653 [77]. LongHam pipeline can be found in github: https://github.com/Gabaldonlab/longHam [78].

## Abbreviations

aa: Amino acids
BRH: Best reciprocal hit
GO: Gene Ontology
GPI: Glycosylphosphatidylinositol
SP: Signal peptide
TEs: Transposable elements
