# Supplementary material for: Chromosome-level assemblies from diverse clades reveal limited structural and gene content variation in the genome of Candida glabrata
Source: BMC Biol. 2022 Oct 8;20:226. doi: 10.1186/s12915-022-01412-1 (PMC9548116; doi:10.1186/s12915-022-01412-1)
Supplement: Supplementary file 1 — Additional file 1: Fig S1. Comparison of reference assemblies. Fig S2. Circos plots representing structural variation among strains. Fig S3. Dotplot representing improvements in assembly of strain CAS08-0016. Fig S4. Barplot representing origin of accessory families. Fig S5. Graphical representation of the adhesin detection pipeline. Fig S6. Gains and losses of adhesin families in the C. glabrata strains. Fig S7. Summary of the LongHam pipeline. Fig S8. Example of an adhesin gene cluster. Fig S9. Schematic representation of the manual curation process to validate accessory genes. [file 12915_2022_1412_MOESM1_ESM.pdf]

Supplementary figures

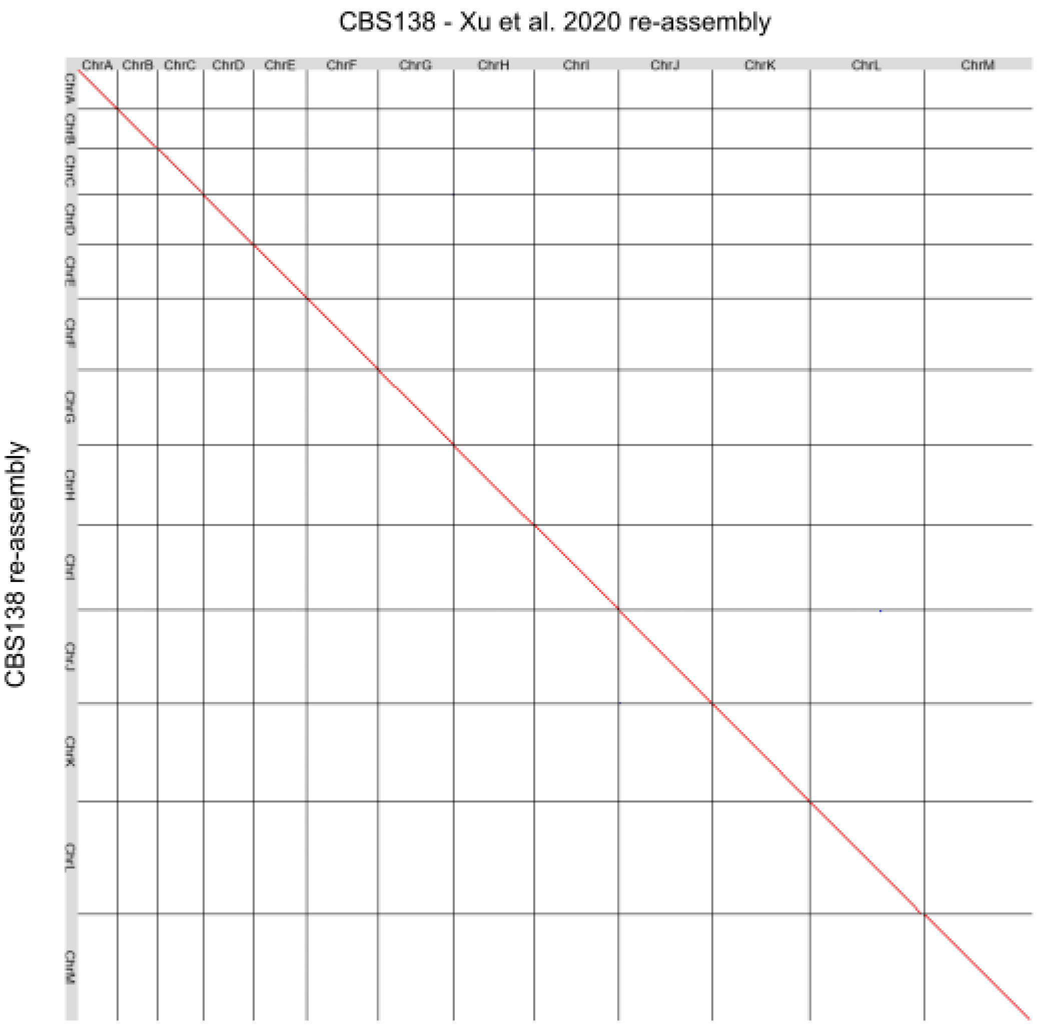

Fig. S1: Dotplot representing the comparison of our re-assembly of *C. glabrata* CBS138 and the one published by Xu et al. [4]. The two assemblies are almost identical except for a small piece of the end of chromosome L that is missing in our assembly.

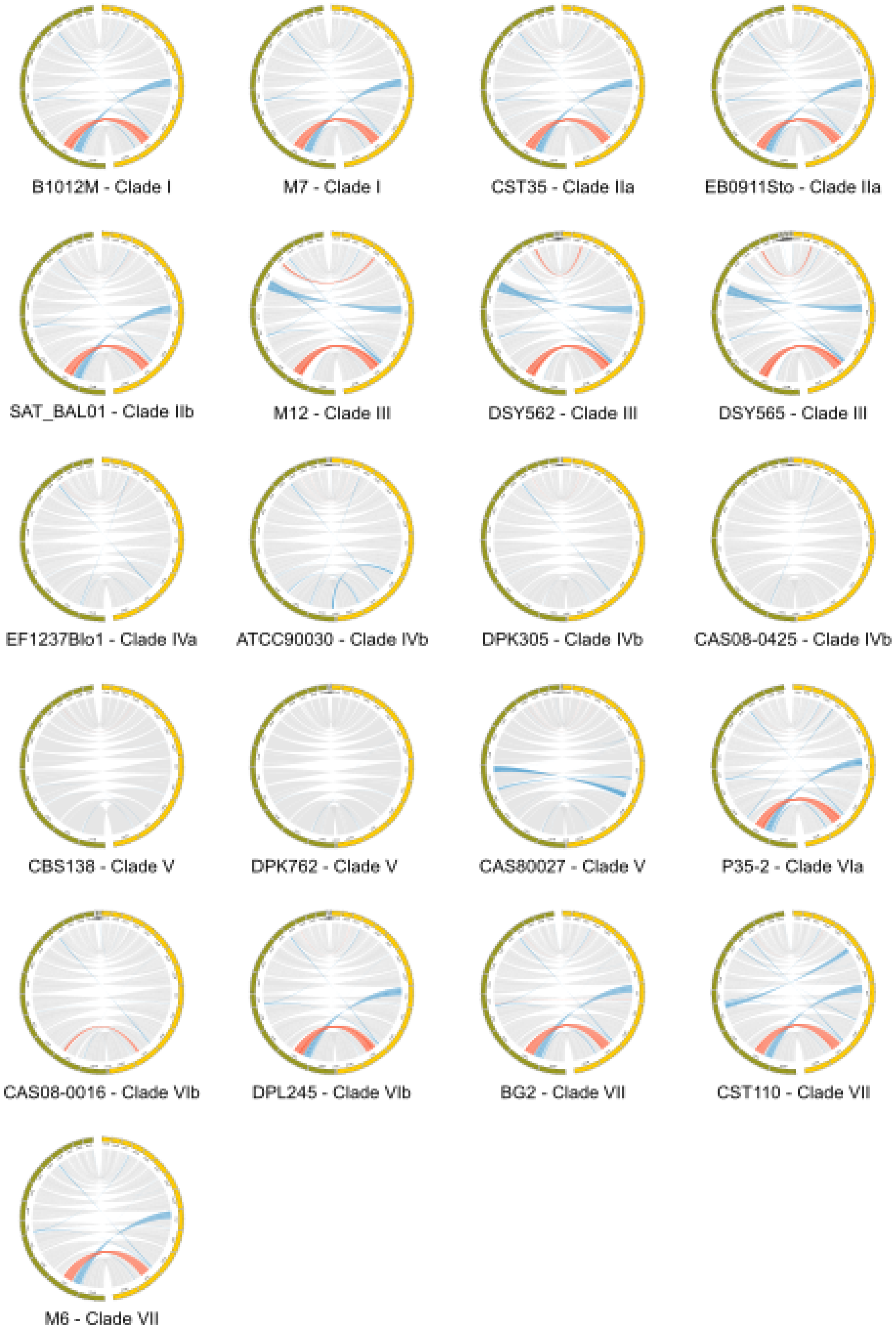

Fig. S2: Representation of the comparison between each strain genome order to the Sanger *C. glabrata* genome. Pictures follow the same format as in Figure 1.

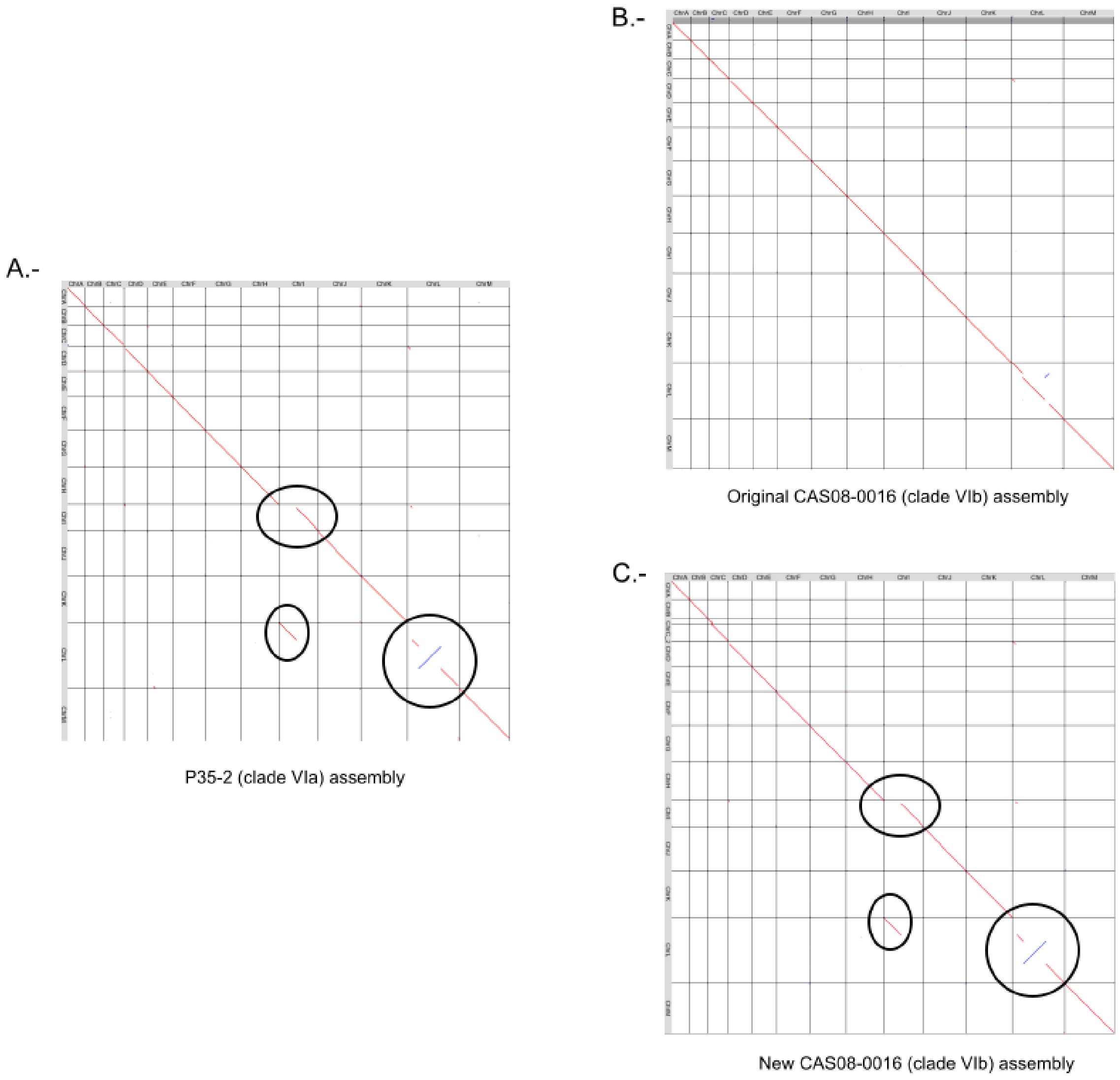

Fig. S3: A.- Dotplot showing homologous regions between P35-2 and CBS138. Black circles indicate the two biggest structural differences found in strains of clades I, II, III, VI and VII: an inversion on chromosome L and a translocation of a piece of chromosome I to chromosome L. B.- Dotplot between the original assembly of strain CAS08-0016 and the reference. The big structural differences between both strains indicated in A are missing in this assembly. C.- Dotplot of the re-assembled CAS08-0016 strain against the reference. In this plot we observe the same major rearrangements seen in strain P35-2.

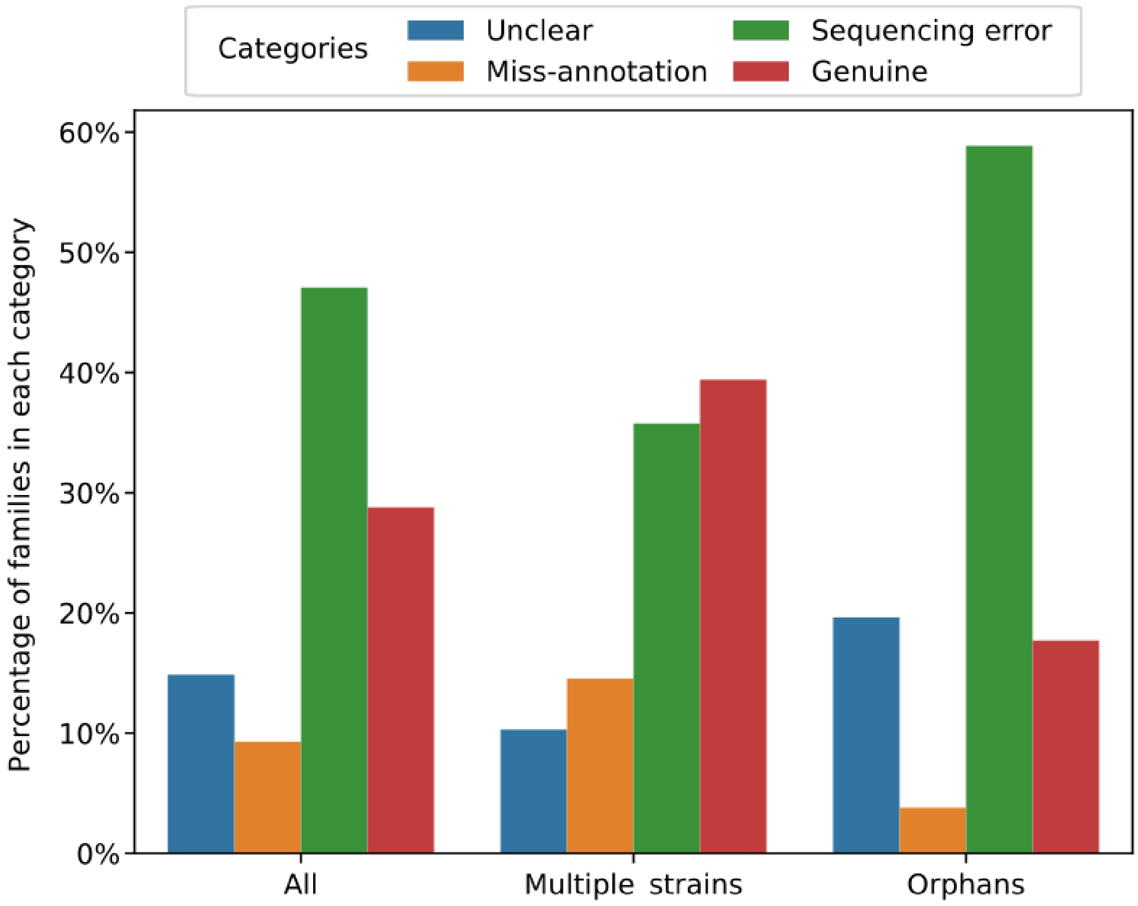

Fig. S4: distribution of accessory families according to their origin in different sets of strains: "All" includes all accessory families, "Multiple strains" includes accessory families present in more than one strain, and "Orphans" includes only families present in a single strain.

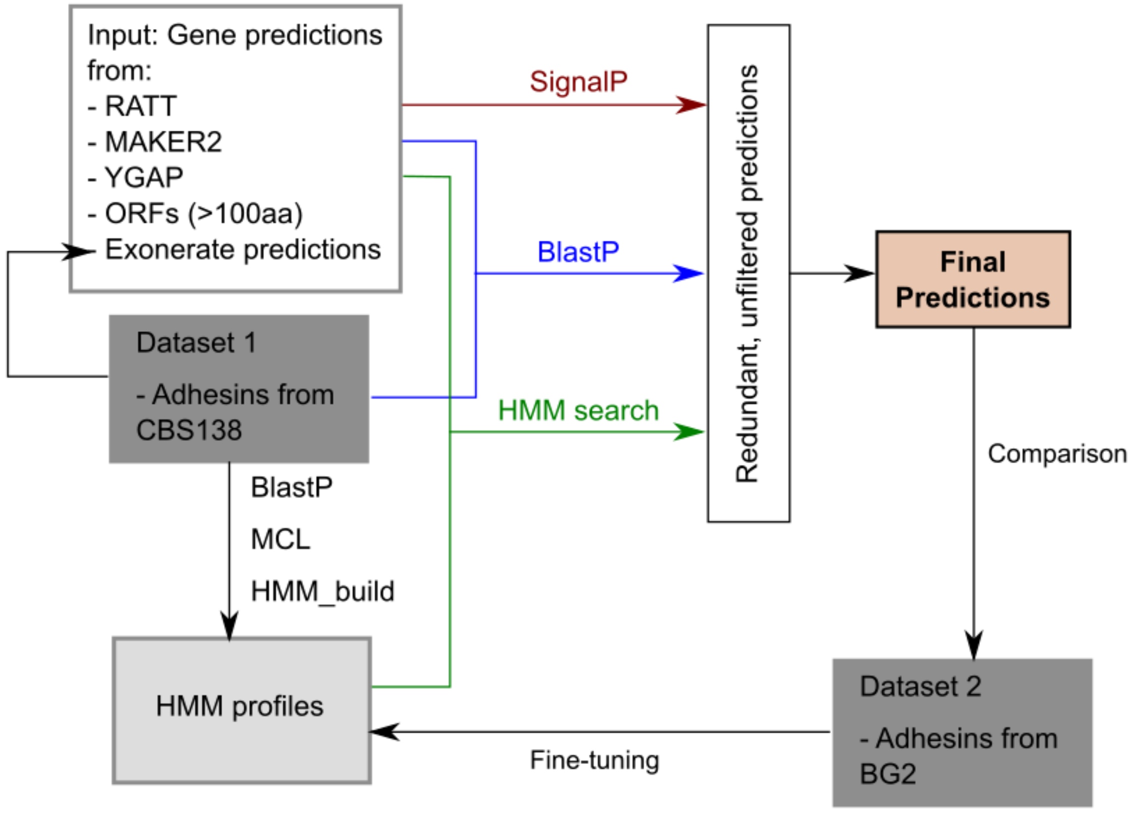

Fig. S5: Graphical representation of the pipeline designed in this study for adhesin prediction.

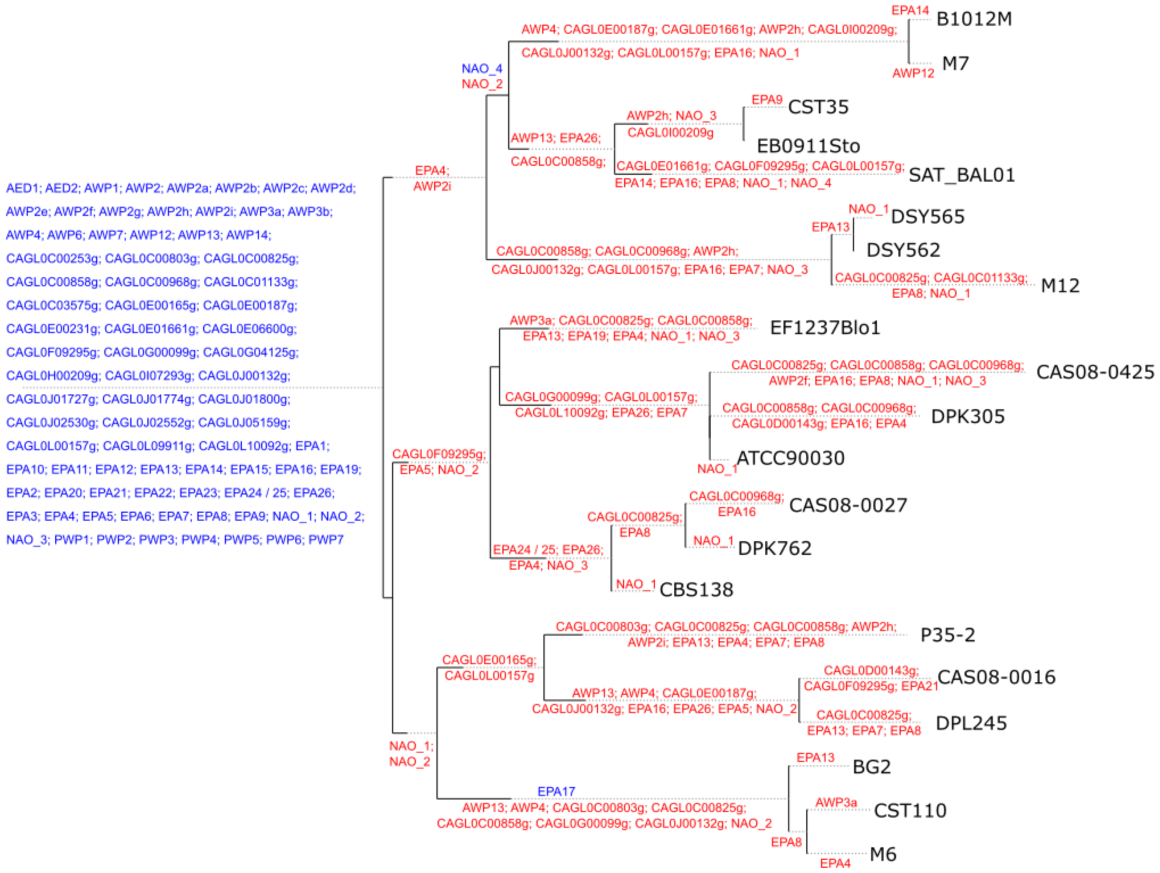

Fig. S6: Gains and losses of adhesin families in the *C. glabrata* strains. Blue numbers represent gains whereas red numbers represent losses.

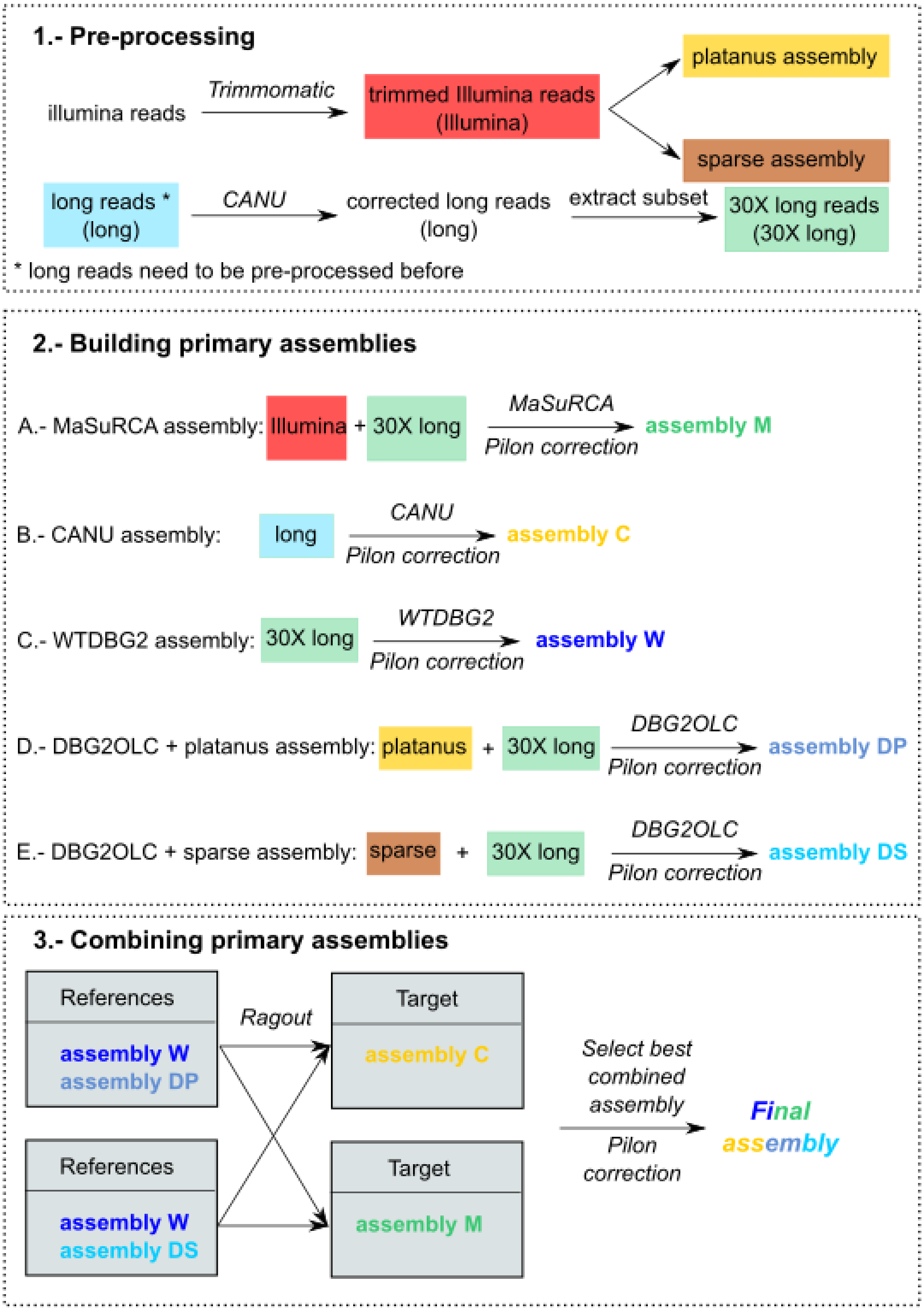

Fig. S7: summary of the LongHam pipeline, used to reconstruct the assemblies of twelve strains of *C. glabrata.* The pipeline is divided into three parts represented by the three dashed rectangles in the figure. The first part (top) includes the pre-processing of short and long reads and the two Illumina based assemblies. Each of the items is marked in a different colour consistent with the colours in the second square. Long reads can be used in two different ways, either using the full dataset (light blue square) or taking the subset of longest

reads trimmed until they have a depth of coverage of 30X (green square). The second part (middle) includes the different primary assemblies, combining different kinds of information obtained from the first step. The produced assemblies are coloured, and the colours are consistent in the third part where they are combined in different ways using Ragout. The final step (bottom) consists of the building of a hybrid assembly made of different primary assemblies. The best combination of primary assemblies is chosen by minimizing the number of scaffolds and maximizing N50.

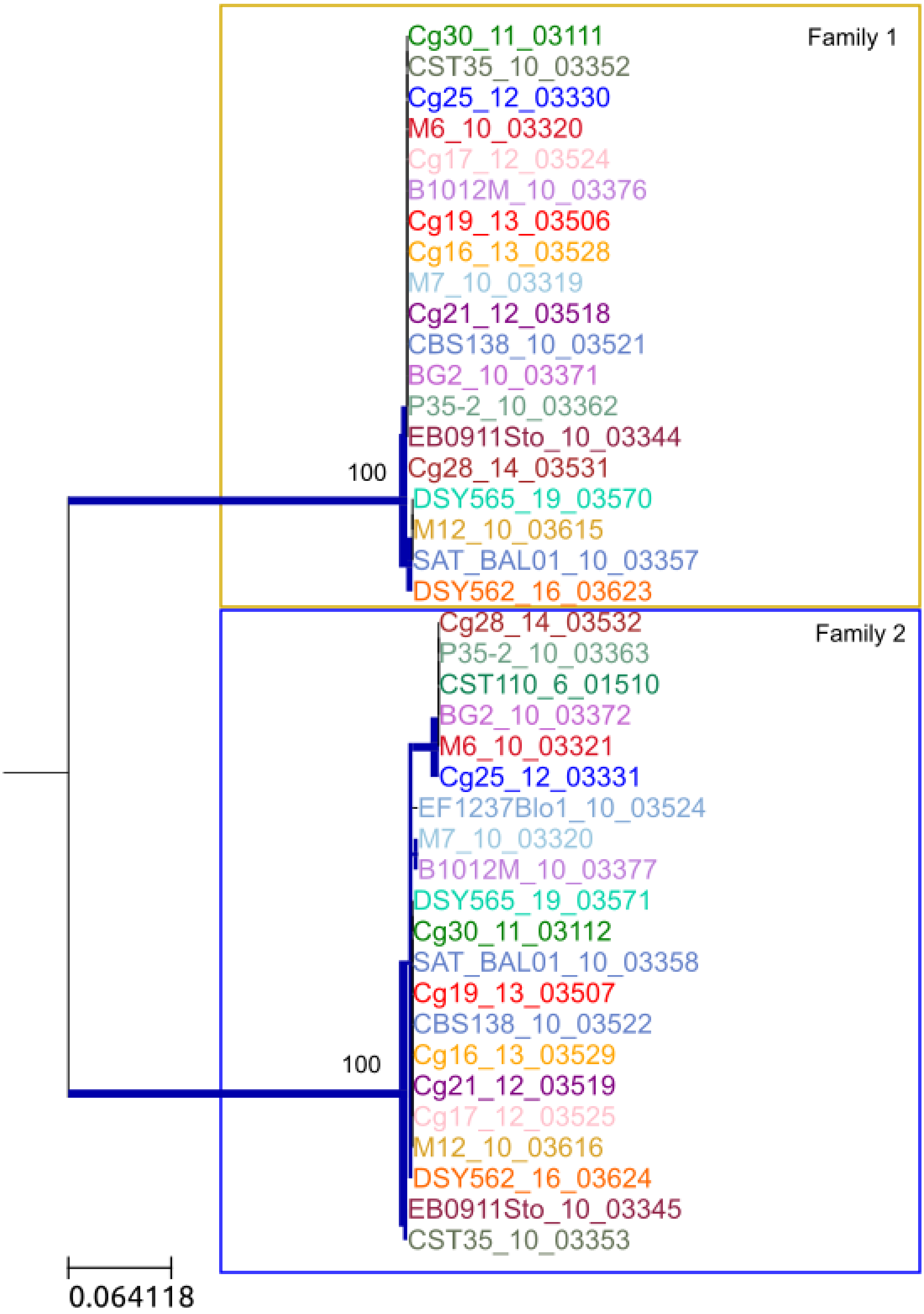

Fig. S8: Example of a phylogenetic tree of an adhesin cluster which is then separated into two families.

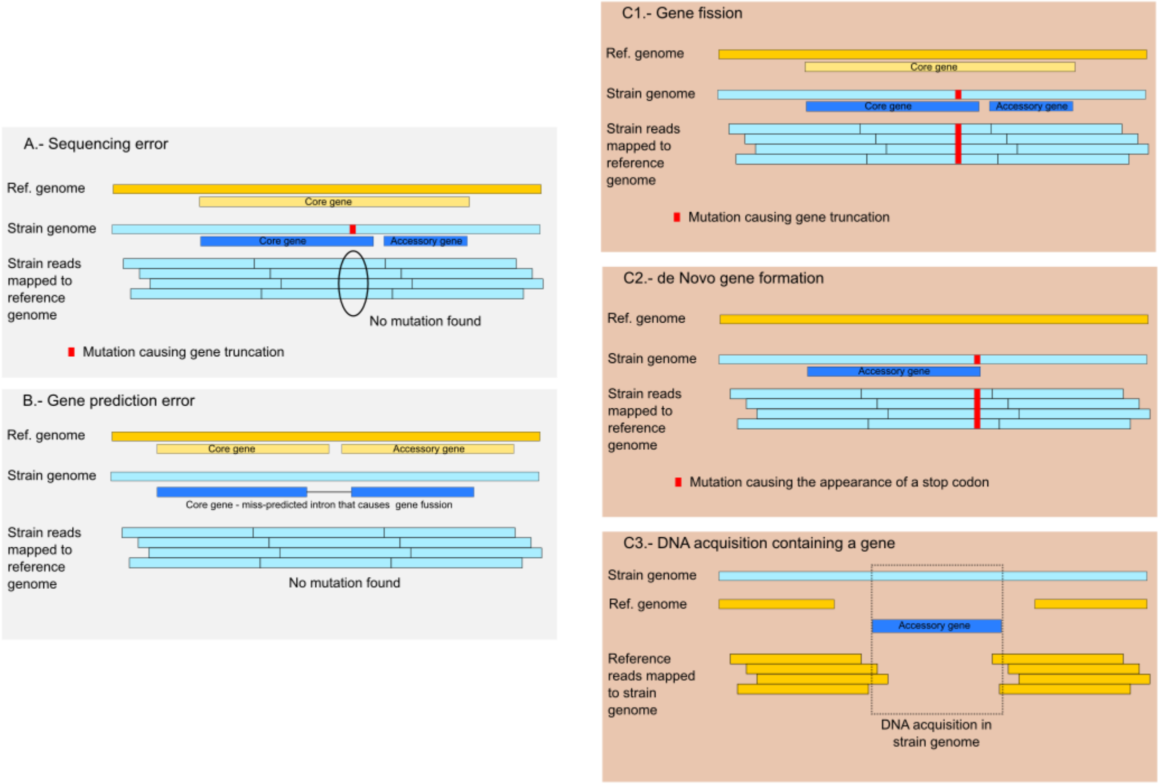

Fig. S9: Schematic representation of the manual curation process to validate accessory genes. Each accessory gene family is checked to assess their origin. If the accessory family results from a mutation or an indel, then Illumina read mapping is checked to see whether the mutation is supported. If a mutation is not the cause of the accessory gene but reads are mapping to the region where the gene should be, then gene predictions are scanned. A.- Shows an example where read mapping does not support an indel, therefore classifying it as sequencing error. B.- Shows a gene prediction where two genes were fused together without any apparent mutation or indel. C1.- Shows an example of how a genuine accessory family is detected, with a mutation supported by read mapping that splits an ancestral gene in two. The large family is classified within the core genome and the smaller gene will proceed to form the accessory family. C2.- Indicates how we detected de novo gene formations. A mutation that causes the appearance of a stop codon and creates an open reading frame where there was nothing in other strains. C3.- Indicates how an accessory family is detected when DNA has been acquired. Reads from the reference strain mapped onto the strain genome do show a gap where the gene is located.
