# Supplementary material for: Chromosome-level assemblies from diverse clades reveal limited structural and gene content variation in the genome of Candida glabrata
Source: BMC Biol. 2022 Oct 8;20:226. doi: 10.1186/s12915-022-01412-1 (PMC9548116; doi:10.1186/s12915-022-01412-1)
Supplement: Supplementary file 3 — Additional file 3. Chromosome-level assemblies from diverse clades reveal limited structural and gene content variation in the genome of Candida glabrata. [file 12915_2022_1412_MOESM3_ESM.pdf]

Additional file 3: Chromosome-level assemblies from diverse clades reveal limited structural and gene content variation in the genome of Candida glabrata

Supplementary text **1**

Legends for supplementary tables **3**

References **4**

Supplementary text

Detailed analysis of relevant adhesion gene families.

The most common type of adhesins are the EPithelial Adhesins (EPA). We found a total of 25 EPA adhesin orthogroups in our analysis, though not all adhesin orthogroups were found in all strains and in some of the strains there were duplications (See main text and Figure 4).

Out of the 25 EPA orthogroups, ten were present in all 21 strains and always in single copy (*EPA1*, *EPA2*, *EPA3*, *EPA10*, *EPA11*, *EPA12*, *EPA15*, *EPA20*, *EPA22*, and *EPA23*). Additionally, *EPA6* was found in all strains but with duplications. We searched whether this effect could be caused by gene conversion events where *EPA7* was replaced by *EPA6* in some strains. *EPA6* and *EPA7* are found in three different loci across our strains: at the left (CL) and right (CR) arms of chromosome C and at the left arm of chromosome E (EL) (Figure 5). The average nucleotide identity between *EPA6* and *EPA7* is 92.9%. On the other hand, for strains with two copies of *EPA6*, the nucleotide identity is 99.6%, showing that the assignment of the two copies of *EPA6* is unlikely to be a phylogenetic artifact. The translocation and gene conversion events affecting *EPA6* and *EPA7* may be involved in pathogenesis, as *EPA6* and *EPA7* have been shown to be expressed during biofilm formation [39].

A similar scenario is found for *EPA4* and *EPA5*, where most strains with *EPA5* duplicated do not have *EPA4*. However, in this case it is difficult to establish whether this is the result of a miss-assembly, the introduction of SNPs in some of the strains, or gene conversion. The two EPA genes are located at the very end of chromosome I, one next to the other, which could indicate that they emerged through segmental duplication and where genetic proximity favored gene conversion. Nucleotide identity between the copies is 100% with. only a deletion of a single nucleotide in *EPA4* in BG2 and CST110. *EPA24* and *EPA25* were joined in the same adhesin orthogroup due to absence of a clear separation in the tree. Their location

on two different chromosomes makes it, in this case, easier to distinguish between the two copies. *EPA24* is found on chromosome A and *EPA25* on chromosome C in strain BG2. Assuming gene order is conserved in this case, clade VII, clade IIa and clade VIa have both, *EPA24* and *EPA25.* Clade I, clade IIb and clade III only have *EPA25* whereas clade IVa, clade IVb, clade VIb, only contain *EPA24*. Clade V has lost both copies. The only duplications that cannot be directly linked to the absence of another EPA gene are the duplications of *EPA8* for strains DSY562 and DSY562 and the duplication of *EPA9* in P35-2.

Some other EPA genes appear in few strains, for instance, *EPA17* is found exclusively in clade VII. *EPA26* is present in most clades except for clade IIa, IIb, V, and DPL245 (clade VIb), whereas *EPA16* does not have a clear clade distribution. We also searched for other inconsistencies related to genomic location of the remaining EPAs and that could not be attributed to the large chromosomal rearrangements already described. We found that for strain EF1237Blo1 (clade IVa), *EPA26* moved from chromosome C to chromosome A. *EPA14a* and *EPA14b* have been collapsed in all our genomes into a single locus, some of them found on chromosome M and in some others on chromosome L. Unlike in previous cases, there is no relation to clade distribution. Most EPA genes are found in telomeric and subtelomeric locations, making it difficult to properly assemble them even when using long reads. Limiting the analysis to the N-terminal regions has allowed us to map presence / absence of the different EPA genes. Although we cannot completely discard that, in some cases, the genomic location of EPA genes may be the result of miss-assemblies, the overall high consistency between location and clade distribution supports our inferences.

AWP proteins have been found as components of the cell wall of *C. glabrata,* indicating their potential importance in adhesion. This group of adhesins is sub-divided into different clusters, of which the largests, with 11 members, is cluster V. The most well known genes are *AWP2* and *AWP4* which encode adhesins recently identified in the cell wall of hyper adherent cells of *C. glabrata* under planktonic conditions whereas other AWP genes were only found in strains that were prone to grow on polystyrene [40]. This suggests that these proteins could be involved in adhesion to medical equipment. We assessed the presence of these genes in the strains included in this analysis. In this case we followed the naming convention proposed in [40] which identifies *AWP2, AWP4* and *AWP2a* to *AWP2i* as members of the cluster. While most orthogroups were found in single copy in all strains some clades were missing some of the genes. *AWP2h* and *AWP2i* were the most sparsely distributed of the 10 *AWP2* orthogroups. They are both missing in clades I, IIa, III and VIa. Additionally *AWP2i* is also lost in clade IIb. Their genomic location is conserved except that *AWP2h* moved from chromosome F to chromosome M in clades VIb and VII. Additionally *AWP4* was lost in clades I, VIb and VII. This indicates that clade I has lost the largest amount of AWP genes belonging to cluster V. The most interesting case is found in gene *AWP2d* (also known as *AWP11*). This gene is found in all strains in chromosome J, except for CST110 that has it in chromosome F due to a large translocation found in this strain. Additionally some clades contain duplications, which does not affect CBS138 or BG2. So, clade I has a second copy of the gene in chromosome M and clades IIa, IIb and VIb have copies in chromosome H. Looking at the phylogenetic tree based on nucleotides more closely it appears that the

duplication that led to the duplicates was ancestral to all strains included in this analysis and losses affected differently members of different clades. So, for instance, members of clade III, VIa and VII retained one copy whereas members of clades IV and V retained another one. Note that this points to CBS138 and BG2 having two slightly different copies of AWP2d. Also, in all cases it was the second copy that changed chromosomes, probably in three independent instances, once to chromosome M (clade I) and twice to chromosome H (clades IIa and IIb in one step and clade VIb in another one). Other AWP orthogroups were found to be complete such as *AWP1, AWP3b, AWP6, AWP7* and the recently described *AWP14* [7]. *AWP13* was missing in clades IIa, IIb, VIb and VII. *AWP3a* was only missing in two independent strains: EF1237Blo1 (clade IVa) and CST110 (part of clade VII) and *AWP12* was missing in M7 (part of clade I).

Legends for supplementary tables

Table S1: Assembly statistics for the re-assembly of the genomes from Arastehfar et al. [20]. Columns indicate strain name, genome size in Mb, number of scaffolds and N50, which represents the length of the shortest sequence found within the group of longest sequences that represent 50% of the genome.

Table S2: List of rearrangements when compared to the *C. glabrata* sanger reference strain. First column indicates the name assigned to the rearrangement. Second column indicates the strain names. Third, fourth and fifth columns indicate the chromosome in the sanger reference genome and the start and stop positions of the rearrangement, respectively. The next three columns indicate the same information for the strain genome.

Table S3: Summary of the results of the gene annotation for the 21 *C. glabrata* strains. The first column indicates the name of the strain, the second column the total number of predicted genes for each strain, the third column the number of genes present in the sanger CBS138 reference genome, and the last column the percentage of the reference genome with a homolog.

Table S4: Complete list of orthogroups as determined by orthofinder. The first column includes the name of the orthogroup, the second includes whether they are part of the core genome or the accessory genome and the third column indicates the results for the manual curation of the accessory genome divided into: genuine (the accessory orthogroup exists even though it may not be functional), sequencing error / miss-predicted or unclear (we were not able to verify the orthogroup due to its location within a repetitive region). Remaining columns indicate the genes belonging to the orthogroup for each strain.

Table S5: List of chromosomes that were substituted in the Ragout assembly by equivalent contigs of primary assemblies.

Table S6: List of predicted adhesins for the 21 *C. glabrata* strains. First column indicates the strain name, the second column the systematic ORF name, the third column the chromosome or scaffold on which the adhesin is located. The following columns indicate the presence (Y) or absence (N) of "VSHITT" and "SFFIT" repeat cores, a signal peptide (SP) for secretion, and GPI anchoring peptide (GPI). Column H indicates the adhesin cluster they belong to. Based on manual curation, column I indicates whether the adhesin is thought to be complete (ok). If not, it indicates whether it is the N-terminal, an internal, or the C-terminal fragment. The final column includes the protein sequence of the adhesin.

Table S7: Phylogenetic trees reconstructed to group adhesins into families. The first column indicates the tree code, the second column summarizes some of the main adhesin families included in each tree, and the third column contains the tree topology in newick format.

Table S8: List of adhesin orthogroups as determined based on phylogenetic analysis. The first column indicates the name of the gene found in the CBS138 or BG2 genome. The second column indicates whether the adhesin is found in a (sub)telomeric region, and finally there is the list of adhesins belonging to each orthogroup, one column per strain.
